# Small cell neuroendocrine carcinoma of the ureter: A case report and literature review

**DOI:** 10.3892/ol.2013.1757

**Published:** 2013-12-12

**Authors:** JIANG HAI PING, ZHANG XIAO CHEN, QIAN JIONG, YOU QI HAN, XU NONG

**Affiliations:** 1 Department of Medical Oncology, The First Affiliated Hospital, School of Medicine, Zhejiang University, Hangzhou, Zhejiang 310003, P.R. China; 2 Department of Pathology, The First Affiliated Hospital, School of Medicine, Zhejiang University, Hangzhou, Zhejiang 310003, P.R. China

**Keywords:** ureter, neuroendocrine carcinoma, chemotherapy

## Abstract

Small cell neuroendocrine carcinoma arising in the ureter is extremely rare; only a few cases have been previously reported in the literature. The current study reports the case of a 65-year-old female who presented with right-sided back pain. A mass was identified in the right ureter, and a nephroureterectomy was performed. The microscopic examination revealed that the mass was composed of a monotonous population of small cells and that the cells of the carcinoma were positive for cluster of differentiation 56, chromogranin A and synaptophysin. The tumor was diagnosed as a ureteral neuroendocrine small cell carcinoma. The patient returned 4 months later with recurrences in the retroperitoneum. Chemotherapy was administered and following 80 mg/m^2^ intravenous irinotecan on days 1 and 8 and 25 mg/m^2^ cisplatin on days 1–3, every 21 days for 4 cycles, the tumor was considerably smaller. During the regular follow-up examinations, the tumor remained stable.

## Introduction

Neuroendocrine carcinomas consists of a heterogeneous group of neoplasms, which arise from peptide- or amine-producing endocrine cells throughout the body (1). The most frequent primary locations of the tumors are pulmonary. The extrapulmonary counterpart is rarely encountered, and has been identified in the gastrointestinal tract, pancreas, genitourinary tract, lymphatics, thymus and peritoneum (2). In the genitourinary tract, the bladder is the most common primary site of the disease, while tumors originating from the ureter are extremely rare (3). Due to its rarity, little is known concerning the natural history of ureteral small cell neuroendocrine carcinoma. The current report presents a case and systematic literature review on the clinical presentation and treatment of this rare tumor.

## Case report

**Figure 1:**
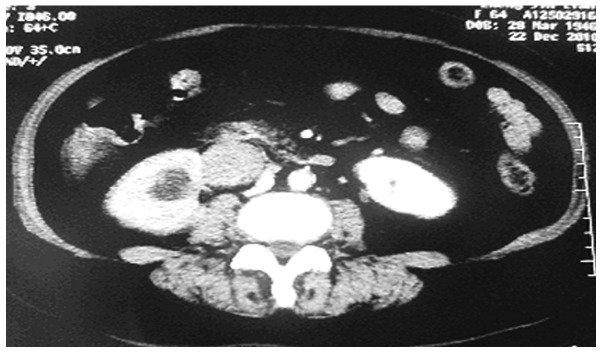
CT scan revealing a mass measuring ~5×4 cm in the right ureter, with light hydronephrosis. CT, computed tomography.

**Figure 2:**
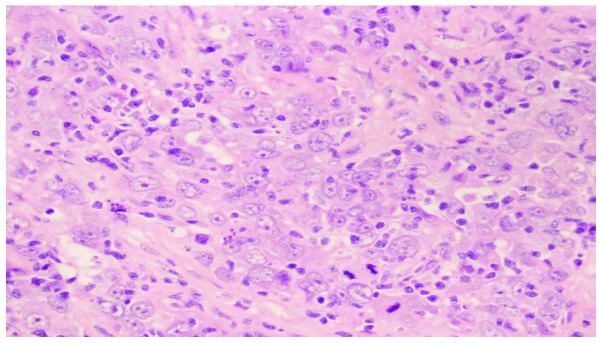
Microscopic examination confirming small cell neuroendocrine carcinoma of the ureter (magnification, ×400).

**Figure 3:**
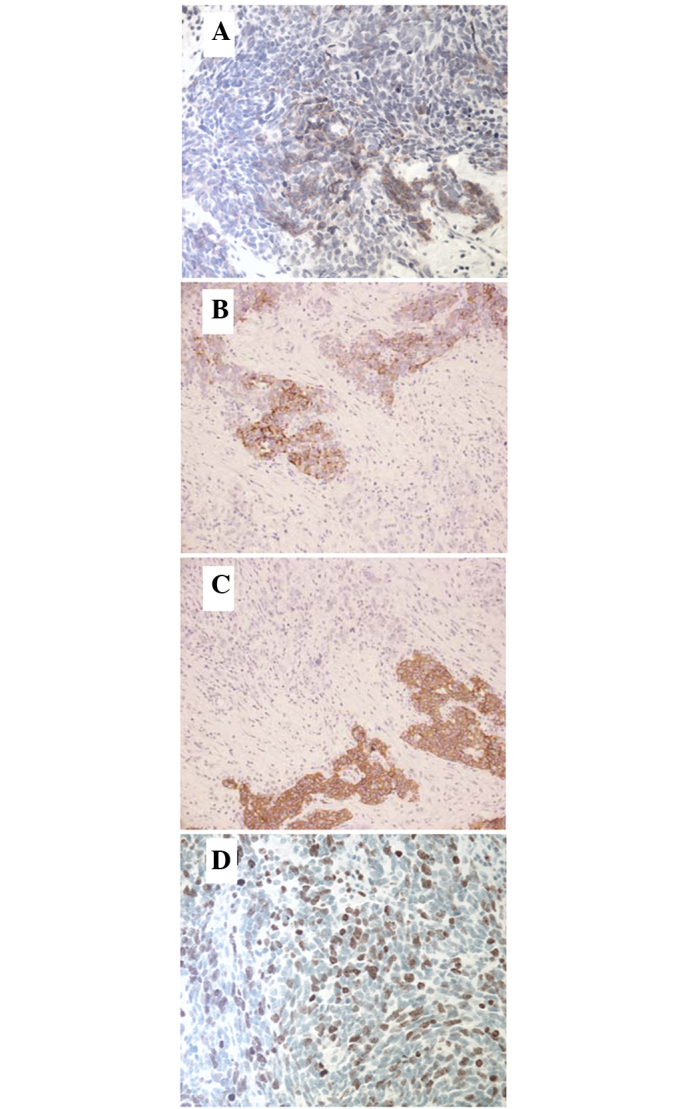
Tumor cells were positive for (A) cluster of differentiation 56 (CD56; magnification, ×400), (B) chromogranin A (CgA; magnification, ×200), (C) synaptophysin (Syn; magnification, ×200) and (D) Ki-67 (magnification, ×400).

**Figure 4:**
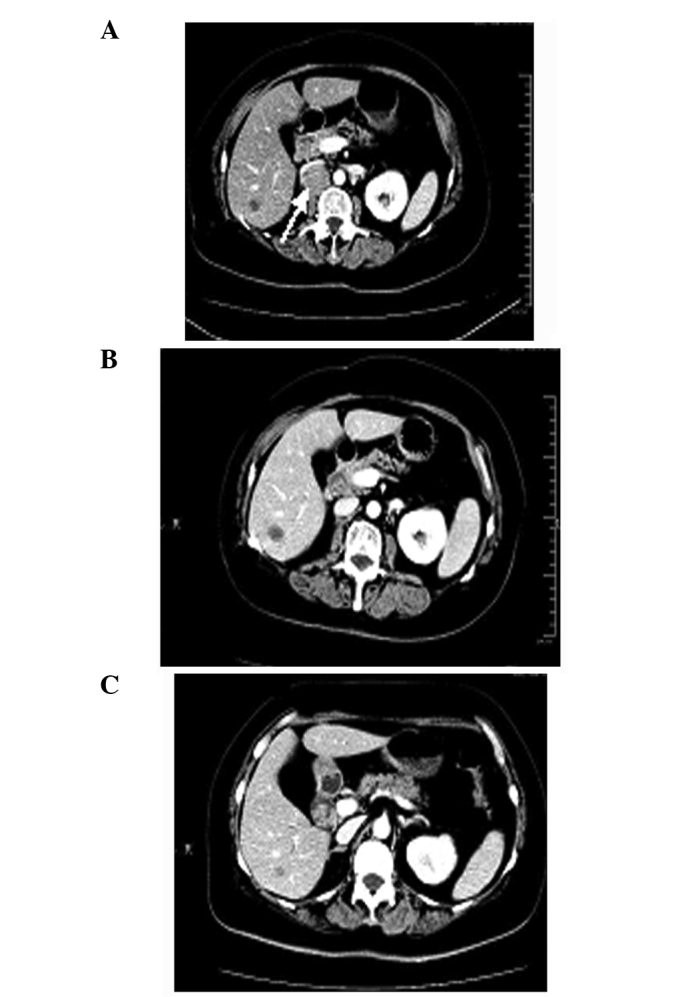
CT scan revealing (A) local recurrences in the retroperitoneum and (B) a considerably smaller tumor following chemotherapy with 80 mg/m^2^ intravenous irinotecan on days 1 and 8 and 25 mg/m^2^ cisplatin on days 1–3, every 21 days for 4 cycles. (C) The tumor was stable during the regular follow-up examinations. CT, computed tomography.

A 65-year-old female presented with right-sided back pain that had been present for one month. The patient had no significant past medical or family history of disease. The physical examination was normal, and laboratory test results showed normal liver function, electrolytes, carcinoembryonic antigen and carbohydrate antigen 19-9. The patient provided written informed consent. A computed tomography (CT) scan of the abdomen revealed a mass measuring ~5×4 cm in the right ureter, with light hydronephrosis (Fig. 1). A chest CT was also reviewed and no primary or metastatic lung lesions were revealed. The patient underwent a right nephroureterectomy and a mass was found within the wall of the right ureter, with grossly negative surgical margins. The microscopic examination showed that the tumor was composed of small cells (Fig. 2). The immunohistochemical staining for the tumor cells was positive for cluster of differentiation (CD)56 (Fig. 3A), chromogranin A (CgA; Fig. 3B) and synaptophysin (Syn; Fig. 3C). Moreover, the tumor expressed high mitotic activity of >20 mitoses per 2 mm^2^, and the Ki-67/MiBi index was 67% (Fig. 3D). The patient was diagnosed with small cell neuroendocrine carcinoma of the ureter. The post-operative recovery of the patient was uncomplicated, but the patient returned 4 months later, with CT scans revealing recurrences in the retroperitoneum (Fig. 4A). Chemotherapy was administered and following 80 mg/m^2^ intravenous irinotecan on days 1 and 8 and 25 mg/m^2^ cisplatin on days 1–3, every 21 days for 4 cycles, CT scans showed a considerably smaller tumor (Fig. 4B). During the regular follow-up examinations, the tumor remained stable (Fig. 4C).

## Discussion

Neuroendocrine carcinomas arising from the urinary tract are extremely rare and represent <0.5% of urinary tract tumors. The bladder is the most common location of small cell neuroendocrine carcinomas, whereas they are extremely rare in the ureter (3), with only 24 previous cases found in the literature since the first case in 1986, which was reported by Ordonez *et al* (4). To the best of our knowledge, the present case is the twenty-fifth to be reported and the first case to be reported in the Chinese population.

Due to its rarity, the origin of these tumors remains controversial and warrants further studies. The following four hypotheses have been suggested for the origin of the tumors: i) From the urothelium with neuroendocrine differentiation; ii) from neuroendocrine cells present in the urinary tract; iii) from the entrapped neural crest in the ureter during embryogenesis; and iv) from undifferentiated stem cells that differentiate towards a urothelial or squamous cell lineage (5,6).

These tumors are commonly observed in the sixth decade of life, with a slight female preponderance, as presented in the current case report. Hematuria and pain are the most commonly reported symptoms of the tumor, with only a few patients exhibiting paraneoplastic syndrome by inappropriate antidiuretic hormone secretion, ectopic adrenocorticotropic hormone production and hypophosphatemia. The current patient presented with flank pain only, with no hematuria or endocrine syndrome.

The diagnosis of these tumors depends on their pathology and immunohistochemistry. Histologically, these tumors are rarely pure and are frequently admixed with other components, including transitional cell and squamous cell carcinomas, adenocarcinoma, chondrosarcoma and leiomyosarcoma (7). On light microscopy, these tumors consist of small cells, with prominent nuclei, scant cytoplasm and granular chromatin. In addition, a high mitotic index may be observed. Furthermore, immunohistochemical staining for specific neuroendocrine markers, including CD56, neuron-specific enolase, Syn and CgA, may distinguish neuroendocrine small cell carcinoma from other tumors and be useful for determining the correct diagnosis (8). During the diagnosis, it is important to exclude lung small cell carcinoma metastasis to the ureter, although it is rarely encountered (9).

The staging of urinary tract small cell carcinoma is similar to that of lung small cell carcinoma, which includes the following two stages: i) Limited disease, the tumor may be encompassed within a tolerable radiation therapy port, whether or not the tumor is confined to the primary site, and with or without regional lymph node involvement; and ii) extensive disease, the tumor is spread beyond the locoregional boundaries and exceeds a tolerable radiation therapy port (10).

The clinical courses of these tumors are usually aggressive and median survival is only 8.2 months. There exists a high incidence of early dissemination and a frequent recurrence of these tumors, which may be due to the occult metastasis at initial presentation. Majhail *et al* previously reported that the incidence of relapse is ≤60% (11). In the present case report, the patient returned with recurrences in the retroperitoneum 4 months after surgery.

The optimal treatment of small cell neuroendocrine carcinoma of the ureter has not been well established. A number of clinicians suggest multimodality therapy, including surgery, radiation therapy and adjuvant chemotherapy, which may confer improved survival rates (12). Previously, Ouzzane *et al* (4) reported that the median survival time of patients with upper urinary tract small cell carcinoma was 24 months for those administered with chemotherapy versus 12 months for those who underwent surgery alone, however, no statistically significant differences were identified (P=0.56). Furthermore, patients administered platinum-based chemotherapy appeared to exhibit a higher median survival time than those who were not administered a regimen containing platinum (24 vs. 8 months, respectively; P=0.12). The tumor site, i.e., whether the tumor is in the renal pelvis or ureter, has not been significantly associated with survival (4). In the previously reported 24 cases of ureteral small cell neuroendocrine carcinoma, 8 cases received chemotherapy, and among those, only 3 cases were administered a platinum-based chemotherapy with a platinum and etoposide regimen. In the present case, due to the fatigue of the patient and avoiding the hematological toxicity of etoposide, the patient received 80 mg/m^2^ irinotecan on days 1 and 8 and 25 mg/m^2^ cisplatin on days 1–3, every 21 days for 4 cycles. No serious toxicity was observed and the patient endured treatment. Following the treatment, CT scans showed a considerably smaller tumor (Fig. 3B), and during the regular follow-up examinations, the tumor remained stable. The clinical results indicated that irinotecan and platinum may also be effective for ureteral neuroendocrine small cell carcinoma. To the best of our knowledge, there is only one previous study concerning this combination of therapy for these tumors (13). It is possible that the combination of irinotecan and platinum may be an alternative therapy for ureter neuroendocrine small cell carcinoma in the future, such as that in small cell lung cancer.

However, for the majority of patients with small cell neuroendocrine carcinoma of the ureter, these treatments are not enough to achieve cure and other strategies are required to improve therapy for this lethal cancer. New molecular therapeutic approaches have been previously investigated for these tumors. It has been reported that these tumors involve c-kit expression and platelet-derived growth factor receptor-α (PDGFRA) mutation (14), which may be potential therapy targets.

In conclusion, small cell neuroendocrine carcinomas of the ureter are rare tumors and few cases have been previously reported. Surgery combined with adjuvant chemotherapy may confer improved survival rates. New therapeutic approaches for these tumors, including agents targeting c-kit and PDGFRA, are also currently under investigation.
